# Development and validation of COVID-19 with myocardial injury based on 3 methods

**DOI:** 10.1097/MD.0000000000043142

**Published:** 2025-06-27

**Authors:** Xiaoqian Yu, Liling Wang, Chengzhen Zhang, Xueqiang Zhao

**Affiliations:** a Hospital of Shandong Technology and Business University, Yantai, China; b Department of Pediatrics, The First Affiliated Hospital of Shandong First Medical University & Shandong Provincial Qianfoshan Hospital, Jinan, China; c Department of Anesthesiology, Central Hospital Affiliated to Shandong First Medical University, Shandong, PR China; d Department of Cardiology, The First Affiliated Hospital of Shandong First Medical University & Shandong Provincial Qianfoshan Hospital, Jinan, China.

**Keywords:** clinical utility, myocardial injury, novel coronavirus pneumonia, predictive modeling, risk assessment

## Abstract

Novel coronavirus pneumonia (COVID-19) poses a major threat to human health as a global public health problem. Currently, the morbidity and mortality rate of myocardial injury in COVID-19 patients is as high as 59.6%; however, clinical prediction models for myocardial injury in COVID-19 patients are not well developed. This study used a retrospective analysis to include 1737 COVID-19 patients who attended Thousand Buddha Mountain Hospital in Shandong Province from December 2022 to December 2023. Data collection was performed through a medical big data system, and the patients were randomly divided into a training group (1216 cases) and a validation group (521 cases). In this study, 1-factor logistic regression, optimal subset regression, and least absolute shrinkage and selection operator regression were used to screen risk factors for myocardial infarction, and a prediction model was constructed based on the results of multifactor logistic regression. The predictive efficacy and clinical utility of the model were further evaluated using area under the receiver operating characteristic curve, calibration curve, and decision curve analysis. (1) Predictor variables screened by one-way logistic regression, optimal subset regression, and least absolute shrinkage and selection operator regression were included in multifactorial logistic regression, respectively, and the results all showed that age, history of alcohol consumption, diastolic blood pressure, heart rate, body mass index, and cystatin C were important risk factors affecting the occurrence of myocardial injury in patients with new crowns. (2) receiver operating characteristic curves were drawn based on the risk factors screened and the results showed that the area under the curve for the prediction set was 0.78 (0.75–0.81). (3) The calibration curves show that the model has good accuracy, with a mean error of 0.02 for both the training set as well as the validation set models. In this study, a myocardial injury prediction model for COVID-19 patients based on clinical parameters was successfully constructed used age, history of alcohol consumption, diastolic blood pressure, heart rate, body mass index, and cystatin C.

## 1. Introduction

Since its first report in December 2019, novel coronavirus pneumonia (COVID-19) has rapidly evolved into a global pandemic caused by severe acute respiratory syndrome coronavirus 2 (SARS-CoV-2), which has a rapid rate of transmission and generalized susceptibility in the population.^[1]^ COVID-19 is not only a respiratory disease, but may also cause systemic multi-organ functional damage that these include acute respiratory distress syndrome, myocardial injury, coagulation disorders, shock, and multi-organ failure.^[2,3]^ Among these complications, myocardial injury is of particular concern, which includes myocarditis, arrhythmia, and heart failure.^[4,5]^ Myocardial injury has a high prevalence in patients with COVID-19 and often has an insidious onset and rapid progression, which may deteriorate into acute left heart failure, intractable shock, or fatal arrhythmia in a short period of time.^[6]^ This cardiac injury is closely related to the high morbidity and mortality rate of COVID-19, which has been shown to be as high as 59.6% in patients with combined myocardial injury,^[7,8]^ much higher than the all-cause mortality rate of 2.3% in the average new coronary patient.^[9]^ Therefore, timely prediction and intervention of myocardial injury is of great significance in reducing the mortality rate of COVID-19 patients. Although studies have explored the possible mechanisms of myocardial injury caused by COVID-19, including direct viral damage to the heart, hypoxia, cytokine storm, and side effects of anti-SARS-CoV-2 drugs,^[10]^ the specific mechanisms are not fully understood. Currently, clinical biomarkers used to predict myocardial injury, such as ultrasensitive troponin, C-reactive protein, calcitoninogen, lactate dehydrogenase, and IL-6, have insufficient specificity, limiting their widespread use in clinical applications.^[11]^ Chen et al demonstrated that 5-hydroxymethylcytosine signaling in circulating free DNA can be used as an early warning of COVID-19 progression and myocardial injury biomarker, but its detection is costly and time-consuming, which makes it unsuitable for large-scale dissemination.^[11]^ In view of this, the aim of this study was to construct and validate a novel clinical prediction model for myocardial injury in combination with coronavirus pneumonia by using big data analysis and combining patients’ baseline information and routine laboratory indicators. With this model, we hope to provide clinicians with a more accurate predictive tool to guide the early identification and timely treatment of myocardial injury in COVID-19 patients. In this study, clinical data of patients diagnosed with COVID-19 between January 2020 and December 2021 were collected using a retrospective cohort study. Through statistical analysis, we screened potential predictors associated with myocardial injury and established a prediction model. Subsequently, we validated the predictive efficacy of the model to ensure its reliability and validity in clinical applications.

## 2. Materials and methods

### 2.1. Data collection

In this study, patients with COVID-19 diagnosed at Qianfoshan Hospital in Shandong Province between December 2022 and December 2023 were retrospectively analyzed through a medical big data system. A total of 1737 patients were included and randomized into a training set (1216 patients) and a validation set (521 patients) based on a 7:3 ratio. General information of the patients including age, history of smoking, history of alcohol consumption, blood pressure (systolic and diastolic), heart rate (HR), height, weight and body mass index (BMI), and clinical biochemical parameters such as indirect bilirubin, alkaline phosphatase, glycine, total bile acid, alanine aminotransferase, cystatin C (cys-C), creatinine, γ-glutamyltransferase, aspartate aminotransferase (AST), retinol-binding protein, sodium (NA), estimated glomerular filtration rate, total protein (TP), potassium, magnesium, and superoxide dismutase. The study was approved by the Ethics Committee of Qianfoshan Hospital, Shandong Province, China, and data desensitization was adopted to protect individual privacy.

### 2.2. Inclusion criteria

Inclusion criteria included: age between 16 and 95 years; positive results for neocoronavirus nucleic acid testing or home self-assessment antigen; and highsensitivity cardiac troponin levels more than 3 times normal.

### 2.3. Exclusion criteria

Exclusion criteria included: severe heart failure or hepatic or renal failure; chronic kidney disease or renal vascular lesions; malignant tumors and their end states; and acute coronary syndromes.

### 2.4. Grouping criteria

Neocoronary combined myocardial injury group: meeting the diagnostic criteria for neocoronary pneumonia and acute myocardial infarction and aged between 16 and 95 years, a total of 598 patients were included and divided into training and validation sets in a 7:3 ratio. Neocoronary uncomplicated myocardial injury group: meeting the diagnostic criteria for neocoronary virus pneumonia and aged between 16 and 95 years, a total of 1148 patients were enrolled and divided into training and validation sets in a 7:3 ratio.

## 3. Methodology

### 3.1. General information collection

With the MeduCloud system, we extracted and collected information about the patient’s age, height, and weight, and calculated the BMI (weight (kg)/height (m)^2^).

### 3.2. Blood pressure and HR measurements

After the patients sat still for 5 to 10 minutes, 3 consecutive measurements of blood pressure and HR were taken using a HEM 7011 blood pressure monitor (Omron Healthcare, Kyoto, Japan), and the mean values were taken as baseline data.

### 3.3. General biochemical indicators

Patients were fasted for 12 hours, and 5 mL of venous blood was collected in the fasting state in the early morning and tested by the Laboratory Department of Shandong Province Qianfoshan Hospital to ensure the quality of the test. Enzyme endpoint colorimetric method was used to detect liver function, kidney function, blood lipids, and blood glucose.

### 3.4. Sample size calculation

According to statistical principles, the sample size should be at least 10 to 20 times the number of independent variables. In this study, there were 22 independent variables, and the sample size was at least 244 to 489 cases, taking into account a dropout rate of at least 10%. Finally 1737 patients were included.

### 3.5. Statistical methods

The study was statistically analyzed using SPSS (version 25.0) and R (version 4.2.1). Data with more than 10% missing values were deleted, and data with <10% missing values were filled in using multiple interpolation. Normally distributed continuous variables were expressed as mean ± standard deviation, and independent samples *t* tests were used for comparisons between groups. Non-normally distributed data were expressed as median (P25, P75) and compared using the Mann–Whitney *U* test. *P* values <.05 were considered statistically significant. Categorical data were expressed as number of cases and percentages and were compared using the chi-square test. Independent variables were screened by one-way logistic regression, least absolute shrinkage and selection operator (LASSO) regression, and optimal subset regression, and multifactorial logistic regression was used to determine the final risk factors to be included in the visual Nomograms model. Model accuracy was assessed by area under the receiver operating characteristic curve and calibration curves, and decision curves were used to further assess model utility

### 3.6. Ethical approval

The study protocol was approved by the Ethics Committee of Shandong Provincial Qianfoshan Hospital to derive data using processing methods such as shielding personal sensitive information. This study was a retrospective analysis. We employed a de-identification method to retrospectively collect clinical data related to patients’ diagnosis and treatment processes. As the research did not interfere with patients’ medical care, obtaining informed consent forms was not required.

## 4. Results

### 4.1. Patient inclusion

A total of 1737 patients were included in this study, including 598 patients with new crown combined with myocardial injury and 1148 patients without combined myocardial injury. The patients were divided into a training group (1216 patients) and a validation group (521 patients) according to a 7:3 ratio.

### 4.2. Modeling

#### 4.2.1. Single factor analysis

The general data of the patients were analyzed for differences by chi-square test, *t* test and *u* test. The results showed that patients in the new crown combined with myocardial injury group had higher age, higher number of history of coronary artery disease, smoking history, and alcohol consumption, faster HR, higher proportion of males, higher levels of cys-C, serum creatinine, AST, and NA, and lower levels of systolic blood pressure, diastolic blood pressure (DBP), and BMI (Table 1).

**Table 1:** Comparison of general information.

	Without MI (n = 810)	With MI (n = 406)	*P*-value
Male sex, no. (%)	62.7	70.0*	<.01
Age-yr	64.53 ± 17.5	72.22 ± 14.68*	<.01
Comorbidity, no. (%)	69.9	74.1*	<.01
Smoking history, no. (%)	21.9	36.4*	<.01
Alcohol consumption, no. (%)	21.9	40.1*	<.01
SBP, mm Hg	137.82 ± 17.30	134.59 ± 22.41*	<.01
DBP, mm Hg	79.00 ± 10.66	72.41 ± 13.74*	<.01
HR, beats/min	84.28 ± 11.37	89.07 ± 15.31*	<.01
BMI, kg/m²	24.53 ± 1.89	24.22 ± 1.43*	<.01
IBIL [mmol/L, M (P_25_, P_75_)]	3.70 (2.00., 6.00)	3.90 (2.30, 6.20)	.47
ALP [U/L, M (P_25_, P_75_)]	68.50 (56.00, 89.00)	69.00 (55.00, 89.00)	.52
CG [U/L, M (P_25_, P_75_)]	1.77 (1.40, 2.33)	1.76 (1.41, 2.34)	.80
TBA [μmol/L, M (P_25_, P_75_)]	3.20 (1.80, 5.40)	3.10 (1.90, 5.60)	.83
ALT [U/L, M (P_25_, P_75_)]	20.30 (12.40, 38.75)	22.25 (13.40, 41.98)	.15
Cys-C [mg/L, M (P_25_, P_75_)]	1.39 (1.02, 2.12)	1.59 (1.08, 2.47)*	<.01
Crea [μmol/L, M (P_25_, P_75_)]	77.00 (58.00, 109.00)	85.50 (61.75, 131.07)*	<.01
GGT [U/L, M (P_25_, P_75_)]	33.00 (20.30, 57.08)	36.20 (21.00, 65.40)	.28
AST [U/L, M (P_25_, P_75_)]	21.50 (15.08, 32.10)	24.15 (15.80, 39.40)*	<.01
RBP [mg/L, M (P_25_, P_75_)]	38.00 (26.75, 51.04)	39.00 (25.00, 52.93)	.97
TP (g/L)	58.66 ± 7.00	57.17 ± 7.41**	.03
K^+^, mmol/L	4.10 ± 0.58	4.16 ± 0.55	.50
MG^+^, mmol/L	0.92 ± 0.14	0.95 ± 0.14	.61
SOD, U/L	116.08 ± 24.67	111.32 ± 24.11	.58

ALP = alkaline phosphatase, ALT = alanine aminotransferase, AST = aspartate aminotransferase, BMI = body mass index, CG = glycine, Crea = serum creatinine, cys-C = cystatin C, DBP = diastolic blood pressure, GGT = γ-glutamyltransferase, HR = heart rate, IBIL = indirect bilirubin, K = serum potassium, MG = serum magnesium, MI = myocardial injury, RBP = retinol-binding protein, SBP = systolic blood pressure, SOD = superoxide dismutase, TBA = total bile acid, TP = total protein.

* *P* < .01.

** *P* < .05.

#### 4.2.2. Multifactor regression analysis

Variables that were significant in the univariate analysis (gender, age, smoking history, drinking history, systolic blood pressure, DBP, HR, BMI, cys-C, serum creatinine, AST, estimated glomerular filtration rate, TP, NA) were included in the multifactorial regression analysis (Table 2). The results showed that age, history of alcohol consumption, DBP, HR, BMI, and cys-C were significant risk factors for myocardial injury (Table 3).

**Table 2:** Univariate analysis.

	B	*P*-value	95% CI
Male sex, no. (%)	0.33	.01	1.38 (1.07, 1.79)
Age, yr	0.03	<.01	1.03 (1.02, 1.04)
Comorbidity, no. (%)	0.21	.12	1.24 (0.95, 1.62)
Smoking history, no. (%)	0.72	<.01	2.05 (1.58, 2.67)
Alcohol consumption, no. (%)	0.88	<.01	2.40 (1.85, 3.11)
SBP, mm Hg	-0.01	<.01	0.99 (0.99, 1.00)
DBP, mm Hg	-0.05	<.01	0.95 (0.94, 0.96)
HR, beats/min	0.03	<.01	1.03 (1.02, 1.04)
BMI, kg/m²	-0.11	<.01	0.90 (0.84, 0.97)
Cys-C, mg/L	0.17	<.01	1.19 (1.09, 1.29)
Crea, μmol/L	0.01	.02	1.00 (1.00, 1.00)
AST, U/L	0.01	<.01	1.00 (1.00, 1.01)
TP, g/L	-0.03	<.01	0.97 (0.96, 0.99)

AST = aspartate aminotransferase, BMI = body mass index, Crea = serum creatinine, cys-C = cystatin C, DBP = diastolic blood pressure, HR = heart rate, SBP = systolic blood pressure, TP = total protein.

**Table 3:** Multivariate analysis according to univariate analysis.

	B	*P*-value	95% CI
Male sex, no. (%)	-0.10	.53	0.90 (0.65, 1.25)
Age, yr	0.04	<.01	1.04 (1.03, 1.05)
Smoking histroy, no. (%)	0.14	.47	1.16 (0.78, 1.71)
Alcohol consumption, no. (%)	0.76	<.01	2.13 (1.45, 3.14)
DBP, mm Hg	-0.06	<.01	0.94 (0.93, 0.95)
HR, beats/min	0.05	<.01	1.05 (1.04, 1.06)
BMI, kg/m²	-0.14	<.01	0.87 (0.81, 0.95)
Cys-C, mg/L	0.22	.03	1.24 (1.05, 1.47)
Crea, μmol/L	-0.01	.45	1.00 (1.00, 1.00)
AST, U/L	0.01	.06	1.00 (1.00, 1.00)
TP, g/L	-0.02	.10	0.98 (0.96, 1.00)

AST = aspartate aminotransferase, BMI = body mass index, Crea = serum creatinine, cys-C = cystatin C, DBP = diastolic blood pressure, HR = heart rate, SBP = systolic blood pressure, TP = total protein.

#### 4.2.3. Optimal subset regression

**Figure 1.**
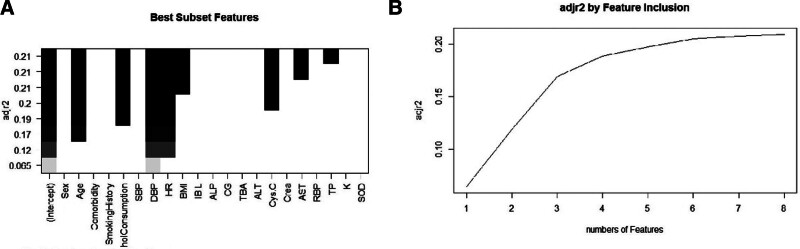
Best subset selection. (A) The result of all possible regression. (B) The number of best subset selection. ALP = alkaline phosphatase, ALT = alanine aminotransferase, AST = aspartate aminotransferase, BMI = body mass index, CG = glycine, Crea = serum creatinine, cys-C = cystatin C, DBP = diastolic blood pressure, GGT = γ-glutamyltransferase, HR = heart rate, IBIL = indirect bilirubin, K = serum potassium, MG = serum magnesium, RBP = retinol-binding protein, SBP = systolic blood pressure, SOD = superoxide dismutase, TBA = total bile acid, TP = total protein.

The optimal subset regression was performed using the Malossian CP minimum, R2 maximum, and Bayesian Information Criterion minimum methods to screen for the optimal combination of independent variables. Age, drinking history, DBP, HR, BMI, and cys-C were identified as the optimal combination of independent variables by the stepwise backward AIC minimization method (Fig. 1).

#### 4.2.4. LASSO regression

**Figure 2.**
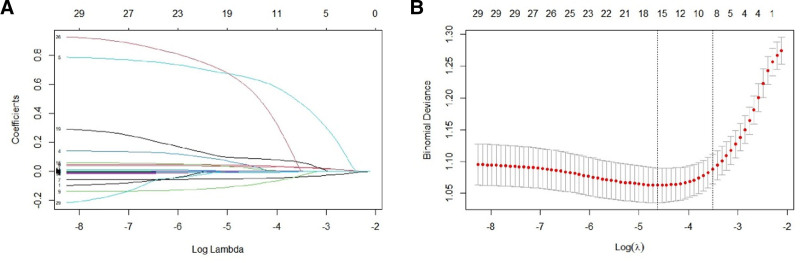
LASSO regression. (A) The result of LASSO regression. (B) The result of cross-validation. LASSO = least absolute shrinkage and selection operator.

Models with fewer number of independent variables were screened by LASSO regression, combined with cross-validation methods to determine the value of λ with the smallest mean square error. The results showed that age, drinking history, DBP, HR, BMI, TP, cys-C, AST, and NA were the independent variables screened by LASSO regression (Fig. 2).

### 4.3. Column line diagram construction

**Figure 3.**
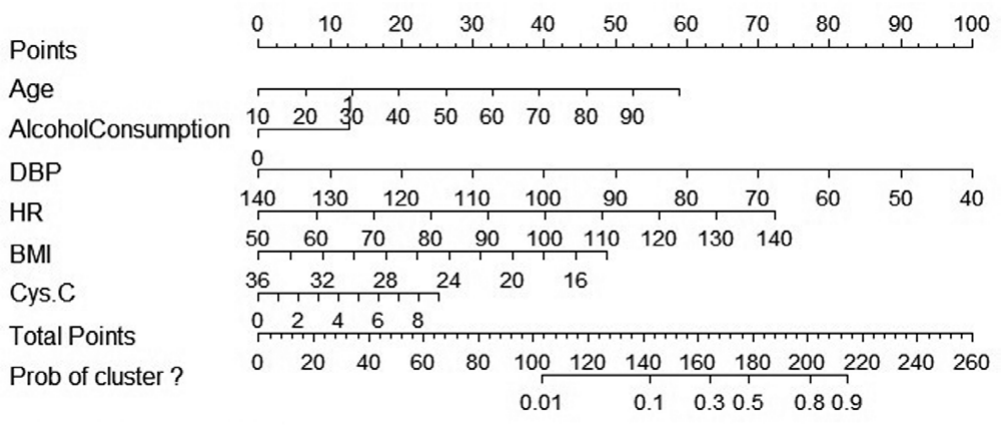
Nomogram plot. BMI = body mass index, cys-C = cystatin C, DBP = diastolic blood pressure, HR = heart rate.

Based on multifactorial logistic regression analysis, the constructed column plot equation was Y = -0.33 + 0.04 × age + 0.78 × drinking history - 0.06 × DBP + 0.05 × HR - 0.14 × BMI + 0.17 × cys-C. The results of the column plot visualization showed that DBP had the highest contribution to the model followed by HR and age, and the contributions of BMI, cys-C, and drinking history had relatively low contributions (Fig. 3).

### 4.4. Model evaluation

#### 4.4.1. Performance evaluation and internal validation

**Figure 4.**
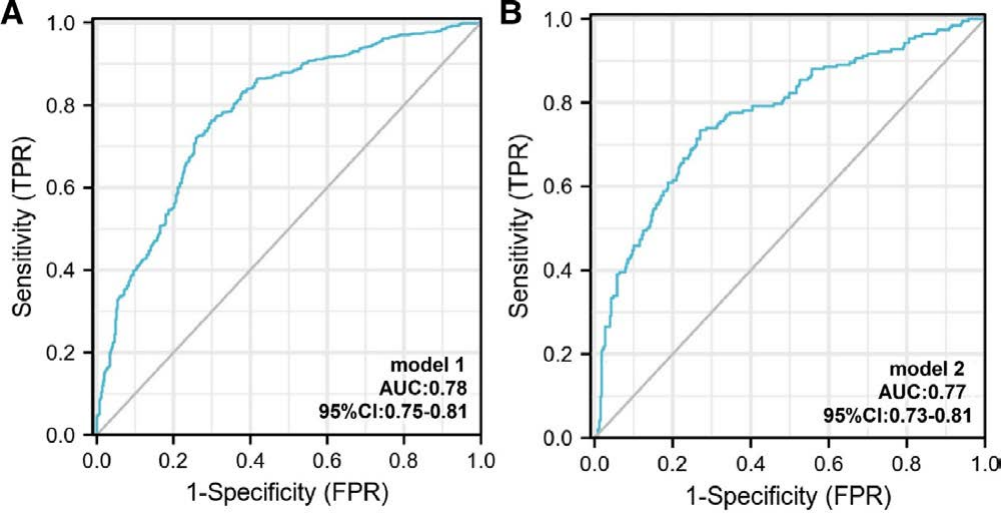
The ROC curve plotted according to the training group and validation group. (A) The AUC of the predictive model was 0.78 (0.75–0.81) with sensitivity and specificity of 76.2% and 70.2% in the training group. (B) The AUC of the predictive model was 0.77 (0.73–0.81) with sensitivity and specificity of 73.4% and 72.9% in the validation group. AUC = area under the curve, ROC = receiver operating characteristic.

**Figure 5.**
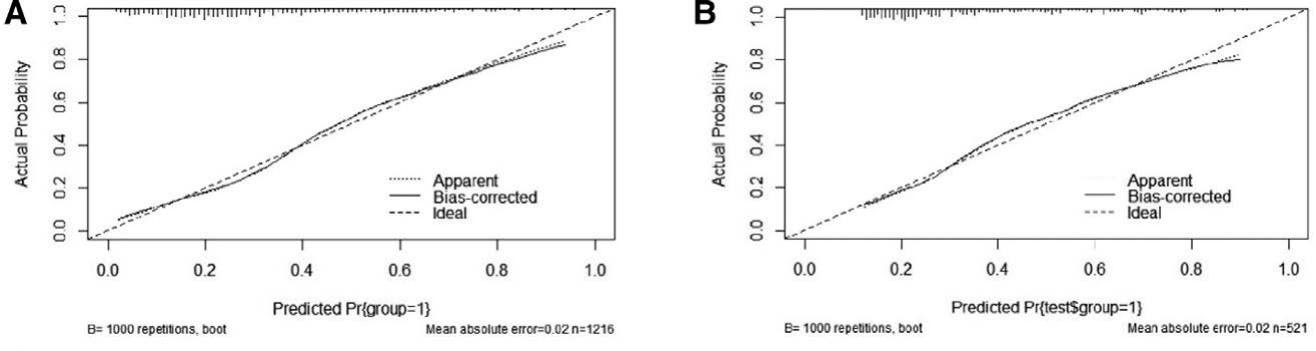
Calibration plot of the training set. (A) The calibration curves were constructed separately for the training group. (B) The calibration curves were constructed separately for the validation group. The predictive models had good calibration with a mean error of 0.02 in both groups.

The area under the curve of the prediction model in the training group was 0.78 (95% CI: 0.75–0.81), with a sensitivity and specificity of 76.2% and 70.2%, respectively, and in the validation group was 0.77 (95% CI: 0.73–0.81), with a sensitivity and specificity of 73.4% and 72.9%, respectively. The calibration curves showed that the model was well calibrated with a mean error of 0.02 (Figs. 4 and 5).

#### 4.4.2. Evaluation of clinical utility

**Figure 6.**
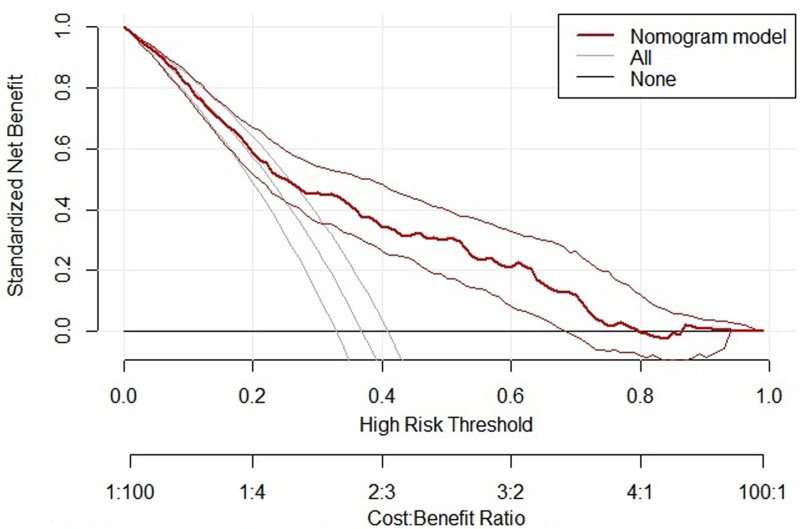
Clinical decision analysis curve of the training set. The DCA curve showed that using the model to predict the risk of MI in patients with COVID-19 produced more benefit when the probability of mycardial injury between 17% to 80%. COVID-19 = coronavirus disease 2019, MI = myocardial injury.

The clinical utility of the model was assessed in the validation group using decision curves. The results showed that the net benefit of using the model to predict the risk of myocardial injury in patients with new crowns was higher than in the absence of the model at prediction probability thresholds ranging from 17% to 80%, suggesting that the model has good clinical utility (Fig. 6).

## 5. Discussion

COVID-19 has become a major public health problem worldwide since its outbreak in late 2019. As an acute respiratory disease, COVID-19 not only causes damage to the lungs, but may also trigger multi-organ dysfunction, especially myocardial damage. According to statistics, about 12% of COVID-19 patients may develop myocardial injury,^[6]^ which significantly increases the risk of mortality in patients. Although several studies have explored COVID-19-associated myocardial injury, the exact mechanism remains unknown, and most of the existing studies focus on prognostic analysis. Relying on the Medo Cloud system and using real-world big data analysis, this study included 1737 patients with COVID-19, focusing on patients’ baseline information and clinical laboratory results. The findings showed that age, history of alcohol consumption, DBP, HR, BMI and cys-C were significant predictors of myocardial injury. We verified the predictive efficacy of the model by multifactorial logistic regression and receiver operating characteristic curve analysis, with an area under the curve of 0.78 (0.75–0.81) and sensitivity and specificity of 76.2% and 70.2%, respectively, which confirmed the accuracy and usefulness of the model.

Current research shows that the possible mechanisms of myocardial injury are complex and varied. First, immune damage in inflammatory state is an important factor. Virus entry into the human body promotes the release of large amounts of inflammatory factors from immune cells,^[12]^ leading to endothelial dysfunction as well as activation of the complement pathway, tissue factor pathway, etc, which in turn leads to diffuse thrombotic microangiopathy, which further promotes thrombosis,^[13]^ and therefore leads to insufficient blood supply to the coronary arteries, and myocardial injury occurs. In addition, the occurrence of inflammation leads to an increase in metabolic demand, which the myocardium is unable to meet, which in turn leads to the occurrence of myocardial injury.^[14]^ Secondly, hypoxia is also a key factor in myocardial injury. Oxygenation as well as hemodynamic levels are worse in neocoronary patients, which in turn leads to elevated pulmonary artery pressure, with cardiac dysfunction, causing myocardial injury,^[15]^ while myocardial injury can lead to ischemia and hypoxia, with reduced ejection capacity of the left heart, aggravated pulmonary stasis, and ultimately the occurrence of an imbalance in the cardiac oxygen supply, which leads to the further occurrence of hypoxia; in addition, hypoxia leads to the buildup of lactic acid in the myocardium, and the vasodilatation and contraction dysfunction, which aggravates the occurrence of myocardial injury. Finally, direct myocardial damage is also an important cause of myocardial injury. ACE2 is an important component of the RAAS system, which can ultimately exert anti-inflammatory and antiproliferative effects by converting ANG II.^[16]^ ACE2 is also an important receptor on the heart, which can effectively regulate the vasoconstrictor and vasodilator interactions in the heart and kidneys by altering the ratio of Ang Ⅱ and Ang (1–7). Balance between vasoconstrictors and vasodilators.^[17]^ It was found that ACE2 acts as an entry receptor for SARS-CoV-2 and SARS-CoV in humans by binding to the viral vesicle membrane protein Spike (S) protein,^[18]^ which may be involved in the pathogenic process of SARS. Currently, autopsies of patients who died from neocoronary combined myocardial injury revealed that their cardiac ACE2 expression was down-regulated and ANG II was elevated.^[19]^ Therefore, it is highly likely that SARS-CoV causes myocardial injury through ACE2.

In this study, we used 3 methods of logistic regression, optimal subset regression, and LASSO regression to screen the independent variables, and the screened independent variables were included in multifactorial logistic regression analyses, and all the results showed that: age, history of alcohol consumption, DBP, HR, BMI, and cys-C were significant predictors of the occurrence of myocardial injury in patients with neocoronary. In our study, we found that the mean age of the population with combined myocardial injury in new crowns was 72 years, which was significantly higher than that of patients without combined myocardial injury. The elderly population generally has reduced immunity, is generally susceptible to infectious diseases caused by viruses or bacteria, etc, and has poor tolerance and long recovery time. A retrospective cohort study conducted by Fan et al^[20]^ found that age is an important risk factor for the development of myocardial injury in patients with new crowns, and that the average age of patients with myocardial injuries was about 70 years old, which is in line with our findings. The relationship between lower DBP and myocardial injury has been confirmed by several studies. A cross-sectional study by Waits et al^[21]^ using the NHANES database showed a very important relationship between low DBP and myocardial injury, especially for patients with DBP below 70 mm Hg [OR (95% CI) 1.40 (1.02, 1.94)] or less. The main mechanism may be that the inflammatory markers produced by new crowns and the resulting oxidative stress cause vasodilatory dysfunction and can directly bind to the ACE2 receptor, affecting the production of angiotensin II and leading to a decrease in DBP, which causes a reduction in coronary perfusion and myocardial ischemia and hypoxia, resulting in myocardial injury. Alcohol consumption as a risk factor for myocardial injury may be related to alcohol-induced vascular endothelial damage and myocardial metabolic disorders.^[22]^ In addition, the relationship between obesity and myocardial injury is complex, and the results of this study showed that patients with lower BMI had a higher risk of myocardial injury, which may be related to the anti-inflammatory effect of adipose tissue.^[23]^ Increased HR is associated with an increased risk of myocardial injury, which may be related to decreased cardiac diastolic time and inadequate coronary perfusion. HR is a dynamic signal dependent on autonomic modulation that regulates the activity of the heart.^[24]^ Taman et al^[25]^ collected data from patients admitted to the ICU due to new crowns for the first 5 days of admission and showed that the R–R interval was significantly shorter in the myocardial injury group than in the non-myocardial injury group on days 1, 2, 3, 4, and 5 of admission to the ICU (*P* < .05). Our findings similarly showed that the risk of myocardial injury in newly crowned patients increased with increasing HR (OR: 1.05, 95% CI: 1.039–1.062). The mechanism may be related to the fact that as HR increases, cardiac diastolic time decreases, coronary perfusion decreases, and myocardial oxygen supply is inadequate, which in turn leads to myocardial injury.

However, this study has some limitations. First, as a single-center cross-sectional study, inferences of causality need to be further validated. Second, changes in highsensitivity troponin were not dynamically monitored, limiting the accurate characterization of viral damage to the myocardium. Finally, the study did not stratify the severity of disease in patients with new crowns, which may affect the overall assessment of the risk of myocardial injury.

## 6. Conclusion

In this study, age, history of alcohol consumption, DBP, HR, BMI, and cys-C were identified as significant predictors of myocardial injury in patients with COVID-19 through big data analysis. These findings provide an important reference for early identification and intervention of myocardial injury. Future studies should further explore the biological mechanisms of these predictors and their applicability in different populations.

## Author contributions

**Data curation:** Chengzhen Zhang.

**Methodology:** Chengzhen Zhang.

**Resources:** Xueqiang Zhao.

**Validation:** Xueqiang Zhao.

**Visualization:** Xueqiang Zhao.

**Writing – original draft:** Xiaoqian Yu.

**Writing – review & editing:** Liling Wang.
